# Effect of ultrasound geometry on the production efficiency of damaged starch: Determining rheology parameters, and non-isothermal reaction kinetics

**DOI:** 10.1016/j.ultsonch.2024.106882

**Published:** 2024-04-20

**Authors:** Reza Roohi, Elahe Abedi, Seyed Mohammad Bagher Hashemi, Masoud Akbari

**Affiliations:** a Department of Mechanical Engineering, Faculty of Engineering, Fasa University, Fasa, Iran; b Department of Food Science and Technology, Faculty of Agriculture, Fasa University, Fasa, Iran

**Keywords:** Ultrasound-assisted damaged starch, Probe size geometry, Thermodynamic analysis, Microstructure analysis, Rheology modeling

## Abstract

Present study investigates the effects of probe size geometry on thermodynamic kinetics, rheology, and microstructure of wheat and tapioca starch. Ultrasound treatment using different probe diameters (20 mm and 100 mm) significantly influenced the gelatinization process. Results showed reduced enthalpy (ΔH) and Gibbs energy (ΔG), indicating enhanced gelatinization efficiency. According to the results, using a 20 mm and 100 mm probe leads to a reduction of 52.7 % and 68.6 % in reaction enthalpy for wheat starch compared to native starch, respectively. Microstructure analysis revealed structural changes, with ultrasound treatment leading to granular fractures and a sheet-like structure with air bubbles. The rheological behavior of the starches is found to exhibit shear thinning behavior, with the Casson model providing the best fit for the experimental data. Moreover, rheology modeling using Herschel-Bulkley and power law models showed increased viscosity and shear stress in larger probes. Numerical simulation data demonstrated that probe size influenced ultrasonic pressure, sound pressure level, and thermal power dissipation density, affecting fluid motion and velocity field components. Moreover, the maximum dissipated power decreases from 8.43 to 0.655 mW/m^3^ with an increase in probe diameter from 20 to 100 mm. The average yield shear stress values are calculated as 3.36 and 3.14 for wheat and tapioca starches, respectively. The larger probe diameter leads to greater entropy increases, with tapioca starch showing a 4.72 % increase and wheat starch a 4.97 % increase, compared to 2.56 % and 3.11 %, respectively, with the smaller probe. Additionally, the Keller-Miksis model provided insights into bubble dynamics, revealing increased pressure and temperature with higher pressure amplitudes.

## Highlights

• Reduced enthalpy implies enhanced gelatinization efficiency in higher probe size.
• Herschel-Bulkley and power law models showed increased viscosity in larger probes.
• Probe size influenced sound pressure level and thermal power dissipation density.
• Non-isothermal kinetics predict the gelatinization process and reaction progress.

## 1 Introduction

Ultrasonication has been associated with the emerging concept of “green chemistry and technology,” which focuses on environmentally friendly applications [1]. The application of ultrasonication for the physical modification of starch offers several advantages. Firstly, it reduces the need for chemicals in the modification process, making it more environmentally friendly. Secondly, ultrasonication can significantly reduce processing time compared to traditional methods. Additionally, ultrasonication provides high selectivity and quality in modifying starch, allowing for precise control over desired modifications. These advantages make ultrasonication a promising and sustainable processing method for starch modification, aligning with the principles of green chemistry and technology [2]. Numerous studies have been conducted to explore the impact of ultrasound on various physicochemical properties of starch. These studies have focused on properties such as structure (depolymerization or polymerization) and functionality mainly, swelling power, solubility, pasting properties, gelatinization temperature, rheology and enthalpy [3], [4]. By gaining a better understanding of these effects, it becomes possible to optimize the use of ultrasonication in starch-related applications while minimizing its environmental impact [5], [6], [7].

While drum drying is a widely used and effective method for producing pre-gelatinized starch, ultrasonication has the potential to serve as an alternative method. Ultrasonication can indeed be used as a pretreatment method for dehydration processes. This is because ultrasonication enhances water diffusivity and the surface area of starch granules, creates a sponge effect within the material, forms microscopic channels, and facilitates forced heat and mass transfer [8]. These effects contribute to improved dehydration efficiency by increasing the mobility of water molecules within the material, facilitating the removal of moisture, and promoting faster drying rates. By utilizing ultrasonication as a pretreatment for dehydration, it is possible to achieve more efficient and effective drying processes, reducing energy consumption and processing time [9].

Thermodynamic and rheological models are crucial in starch processing as they provide a scientific framework for understanding and predicting the behavior of starch under various conditions. Thermodynamic models help in determining the energy changes and stability of starch during processes like gelatinization, while rheological models describe how starch pastes flow and deform, which is vital for consistency and texture in food products [10], [11], [12].

Several articles have explored these models in the context of starch processing. For instance, studies have characterized the thermal and rheological properties of starches from different sources, providing insights into their behavior during heating and cooling. Other research has focused on modifying starch properties to tailor them for specific applications, highlighting the role of models in predicting the outcomes of such modifications [13], [14]. Additionally, computational models have been used to simulate starch digestion, offering a deeper understanding of how processing affects digestibility [15].

These models and the associated research are fundamental to advancing starch processing technology, ensuring that products meet the desired quality standards and functional properties.

This paper aims to assess the impact of ultrasound treatment on wheat and tapioca starches, with a specific focus on analyzing alterations in microstructure, pasting attributes, thermal responses, and rheological behaviors. Moreover, the investigation aims to elucidate the influence of differing probe sizes on the gelatinization kinetics and thermodynamic features of the starches. Employing a combination of numerical simulations and experimental observations, the study seeks to explain the underlying mechanisms involved in ultrasonic treatment and its consequent effects on starch properties. The findings of this research hold potential implications for diverse industrial sectors, notably in the realms of food processing and materials science.

## 2 Materials and methods

### 2.1 Materials

NWS (native wheat starch) and NTS (native tapioca starch), obtained from Fars Glucosine in Shiraz, Iran, were analyzed for their composition. NWS was found to contain approximately 25.3 ± 0.15 % amylose, 5.88 ± 0.08 % moisture, 1.94 ± 0.05 % fat, 0.22 ± 0.05 % ash, and 5.26 ± 0.11 % protein. On the other hand, NTS contained approximately 18.78 ± 0.12 % amylose, 7.64 ± 0.11 % moisture, 0.94 ± 0.08 % fat, 0.27 ± 0.03 % ash, and 6.53 ± 0.10 % protein. The composition analysis was conducted following the approved methods of AACC (2000).

### 2.2 Ultrasound-assisted pre-gelatinized starch

A study conducted by Abedi et al. (2019) utilized ultrasound to produce ultrasound-assisted pre-gelatinized wheat starch (UPWS) and ultrasound-assisted pre-gelatinized tapioca starch (UPTS) [16]. The ultrasonic generator used in the experiment was a UP400S Hielscher model from Teltow, Germany, with a power of 400 W and a frequency of 24 kHz. An immersible probe with on–off pulse durations of 5 s was employed and submerged in a 100-mL cylindrical jacket glass vessel measuring 180 × 180 mm^2^. The temperature of the vessel was controlled within the range of 35–65 °C using a circulating water bath. A total of 50 mL of wheat and tapioca starch (at a concentration of 20 % w/w) were subjected to ultrasound treatment in separate experiments. In the experimental setup, the immersible probe was inserted into at the top of the liquid present in the vessel, allowing the sound vibration to be transmitted to the starch sample. This transmission was facilitated by a titanium alloy rod with a diameter of 20 mm and 100 mm, which had a surface area of 3140 mm^2^ and 28260 mm^2^, respectively. This arrangement ensured efficient transfer of ultrasound energy to the starch samples for the desired effects.

### 2.3 Microstructure, pasting and thermal properties

The microstructure analysis of native and modified starches was performed using scanning electron microscopy (SEM) at an acceleration voltage of 20 kV. The SEM instrument used in this study was the Leica Cambridge model from the UK. The pasting properties of starch samples were determined using a rapid viscoanalyzer (RVA). The RVA instrument, manufactured by Newport Scientific Pty., Ltd., was connected to a personal computer for data analysis. The gelatinization properties of starches were investigated using a differential scanning calorimetry (DSC) instrument. The specific DSC instrument employed in this study was the OIT-5000, manufactured by Sanaf Electronic Co. in Iran. To facilitate data analysis, the DSC instrument was equipped with STAR software.

### 2.4 Governing equations

#### 2.4.1 Acoustic field

The linear Helmholtz equation is chosen to implement the simulation of sound wave propagation and distribution within the domain. This choice is based on the fact that the medium being examined is homogeneous [17].

(1) $$\nabla \left[\frac{1}{\rho }\nabla P\right]-\frac{1}{\rho c^{2}}\frac{\partial^{2}P}{\partial t^{2}}=0$$

where in the above equation, the parameters of ρ, c, P and t denote the fluid density, sound speed, acoustic pressure and time, respectively. As the propagation of the sound is performed through oscillating expansion and contraction waves, it is reasonable to use the transformation of $P\left(\vec{r},t\right)=p\left(\vec{r}\right)e^{i\omega t}$ to reform the acoustic pressure equation from time to frequency domain as:

(2) $$\nabla \left[\frac{1}{\rho }\nabla P\right]+\frac{4\pi^{2}f^{2}}{\rho c^{2}}=0$$

This conversion makes it easier to simulate the flow streaming process since the time needed for formation of the acoustic pressure field is much shorter compared to the velocity field. Consequently, the acoustic pressure field is first calculated in the frequency domain before determining the flow field, and the obtained results are utilized to determine the flow streaming force.

#### 2.4.2 Velocity field

The determination of the flow streaming process and bubble formation is performed using the mixture model. The continuity equation can be formulated as:

(3) $$\frac{\partial }{\partial t}\left(\rho_{m}\right)+\nabla .\left(\rho_{m}\vec{V_{m}}\right)=0$$

where ${\vec{V}}_{m}$ is the fluid velocity and the *m* subscript represents the mixture state. The simulation is based on the assumption of existence of two mass interacting phases of liquid and vapor. So, the mixture velocity and density can be formulated as [18]:

(4) $${\vec{V}}_{m}=\frac{\alpha_{v}\rho_{v}{\vec{V}}_{v}+\left(1-\alpha_{v}\right)\rho_{l}{\vec{V}}_{l}}{\rho_{m}}$$

(5) $${\rho_{m}=\alpha }_{v}\rho_{v}+\left(1-\alpha_{v}\right)\rho_{l}$$

where *v* and *l* subscripts denote vapor and liquid phases.

The momentum equation is expressed as:

(6) $$\frac{\partial }{\partial t}\left(\rho_{m}{\vec{V}}_{m}\right)+{\vec{V}}_{m}\nabla .\left(\rho_{m}{\vec{V}}_{m}\right)=-\nabla P+\nabla .\left[\mu_{m}\left(\nabla {\vec{V}}_{m}\right)\right]+\rho_{m}\vec{g}{+\vec{F}}_{\mathrm{Streaming}}$$

The viscosity ($\mu_{m}$) is determined based on the viscosity of each phase as:

(7) $${\mu_{m}=\mu }_{v}\rho_{v}+\left(1-\alpha_{v}\right)\mu_{l}$$

The streaming force (${\vec{F}}_{\mathrm{Streaming}}$) is calculated based on the developed formulation of Maramizonouz et al. (2021) [19].

#### 2.4.3 Bubble dynamics

In 1980, Keller and Miksis introduced the Keller-Miksis model, which has since become widely used in the study of bubble dynamics in different fluid environments. This mathematical model has proven essential for understanding bubble behavior in various fields, including acoustics, cavitation, and underwater explosions.

The model is based on the assumption that the bubble is a spherical cavity filled with an ideal gas, while being surrounded by a non-compressible liquid medium. It takes into account the influence of surface tension, viscosity, and acoustic pressure on the dynamics of the bubble. The Keller-Miksis model incorporates the Rayleigh-Plesset equation, which describes the growth and collapse of the bubble, and expands upon it by considering the compressible nature of the gas inside the bubble.

By utilizing the Keller-Miksis model, predictions about fundamental bubble phenomena, such as oscillations, growth rates, collapse dynamics, and the generation of pressure waves can be made. However, the accuracy of the model is highly dependent on the assumption of ideal gas behavior within the bubble, and it has limitations due to its neglect of more complex effects of real gases and factors such as thermal conduction [20]. The Keller-Miksis model can be formulated as:

(8) $$\left(1-\frac{\dot{R}}{c}\right)R\ddot{R}+\frac{3}{2}{\dot{R}}^{2}\left(1-\frac{\dot{R}}{3c}\right)=\left(1+\frac{\dot{R}}{c}\right)\frac{1}{\rho_{l}}\left[p_{B}\left(R,t\right)-p_{A}\left(t+\frac{R}{c}\right)-P_{\infty }\right]+\frac{R{\mathrm{dp}}_{B}\left(R,t\right)}{\rho_{l}cdt}$$

where in the above equation, R, c, t, $p_{B}\left(R,t\right)$, $p_{A}\left(t+\frac{R}{c}\right)$ and $\rho_{l}$ are bubble radius, sound speed in liquid, time, interface pressure of liquid around bubble, time-delayed driving pressure and liquid density, respectively. The dot symbol is a representation of time derivative.

#### 2.4.4 Boundary conditions

**Fig.1:**
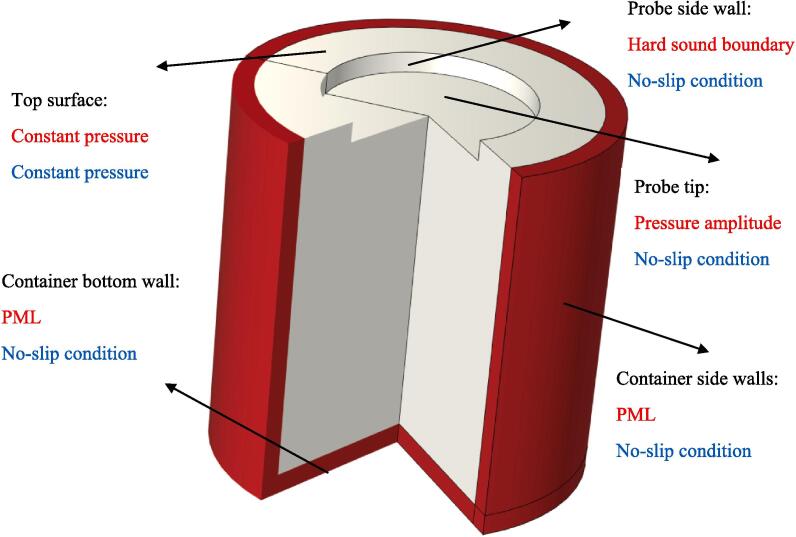
Schematic of the simulated geometry and the implemented boundary conditions.

Fig. 1 shows the computational domain and the applied boundary conditions (the acoustic boundary conditions are shown in red and velocity field boundary conditions are displayed in blue). The acoustic field is subjected to three types of boundary conditions: sound hard boundary, specified pressure, and perfectly matched layer (PML). The PML serves as a substitute method for absorbing boundary conditions. Unlike ordinary absorbing boundary conditions, the PML functions as an artificial additional absorbing layer that expands the original simulation grid. When waves enter the absorbing layer, they are attenuated and decay exponentially. The unique property of a PML is that it enables non-reflecting propagation of waves from a non-PML region to a PML, irrespective of the incident angle. Essentially, the PML behaves like a non-reflecting absorbing material.

In Fig. 1, the PML domain is assigned to regions farther away from the horn tip. The pressure boundary condition is assigned to the tip of the transducer and is calculated based on the sound intensity. The sound intensity is defined as the ratio of the transducer's power to the tip area.

(9) $$I=\frac{P_{\mathrm{UP}}}{A_{\mathrm{probe}}}$$

where I, $P_{\mathrm{UP}}$ and $A_{\mathrm{probe}}$ are the sound intensity, ultrasonic power and area, respectively.

Based on the sound intensity the amplitude of the acoustic pressure (*P_a_*) is determined as:

(10) $$P_{a}=\sqrt{2I\rho c}$$

The remaining surfaces have been designated with the sound hard boundary condition. A sound hard boundary ensures that the sound particle velocity's normal component is zero, as it prohibits any movement in the forward direction.

For the velocity boundary condition, the top surface (except of the horn tip) is assigned as the constant pressure with the atmospheric pressure setting and all other surfaces are set to no-slip boundary condition.

### 2.5 Thermodynamic kinetics and rheology modeling

The non-isothermal kinetics study utilizes a model that assumes *n^th^*-order reaction kinetics, with the reaction rate relying on temperature [21]. In order to determine viscosity under various operating conditions, established models are employed to simulate the relationship between shear stress and shear rate in rheology modeling.

The relationship between the thermodynamic quantities ΔG (Gibbs free energy change), ΔH (enthalpy change), and ΔS (entropy change) in the context of ultrasonicated starches can be understood through the fundamental thermodynamic equation: ΔG = ΔH-TΔS.

The Gibbs free energy equation delineates that the free energy alteration of a process is contingent upon the enthalpic and entropic changes, as well as the temperature at which the process transpires. The implications of these thermodynamic parameters are as follows:

ΔH: This parameter quantifies the starch system’s total thermal energy. An exothermic reaction, characterized by heat release, is denoted by a negative ΔH, whereas an endothermic reaction, which absorbs heat, is indicated by a positive ΔH.

ΔS: This metric gauge the stochasticity or disarray within the starch system. A positive ΔS signifies an escalation in disorder, while a negative ΔS conveys a reduction, implying a more ordered state.

ΔG: This variable is pivotal in ascertaining the spontaneity of a process. Spontaneous processes are signified by a negative ΔG, while non-spontaneous processes are represented by a positive ΔG.

In the ultrasonication of starches, the objective is to alter the structural and functional attributes of the starch. Ultrasonication may precipitate a decline in ΔH, potentially diminishing the energy requisite for the modification process relative to conventional techniques. Concurrently, the entropy, ΔS, may augment due to the disintegration of the starch granules’ orderly structure, engendering a state of greater disorder. This disorder is often associated with the genesis of minuscule channels and the aforementioned sponge effect.

Hence, should ultrasonication result in a reduction of ΔH (owing to enhanced energy efficiency) and an elevation in ΔS (attributable to heightened disorder), the cumulative effect would likely be a shift towards a negative ΔG, intimating that the ultrasonication process might proceed spontaneously under appropriate conditions. Nonetheless, the precise values of ΔG, ΔH, and ΔS are contingent upon the specific parameters of the ultrasonication process, such as the temperature, duration, and the starch variant involved.

### 2.6 Rheology modeling

Fluid modeling plays a fundamental role in rheology, as it allows us to comprehend and predict the flow properties of different substances. A crucial aspect of this process is the determination of fluid viscosity and the establishment of the connection between shear rate and shear stress [22].

Three commonly used rheological models for analyzing starch viscosity are the Herschel-Bulkley, Casson, and power law models. Each model offers insights into different aspects of fluid behavior. The Herschel-Bulkley model incorporates factors such as yield stress and flow behavior index to describe the non-Newtonian nature of the fluid [23].

On the other hand, the Casson model defines the properties of the fluid by presenting a yield stress, followed by a proportionate correlation between shear rate and shear stress. This model is appropriate in situations where there is a noticeable yield stress. Finally, the power law model explains the flow characteristics by taking into account the consistency index and flow behavior index. It assumes an equation in the form of a power law that relates shear rate and shear stress.

#### 2.6.1 Herschel-Bulkley model

The Herschel-Bulkley fluid model is an improvement upon the Bingham model and utilizes experimental data to calculate viscosity of non-Newtonian fluids. It takes into account the consistency index, shear thickening/thinning, and yield stress to establish the relationship between shear stress and rate [24].

(11) $$\tau =\tau_{\mathrm{yield},1}+\varepsilon {\dot{\gamma }}^{\beta }$$

The equation above represents the values of stress (τ) and yield stress ($\tau_{\mathrm{yield},1}$). furthermore, the parameters *ε* and *β* indicate the consistency and flow behavior indexes, respectively.

#### 2.6.2 Casson model

The Casson model serves as a widely recognized framework for evaluating the viscosity of both food and biological substances. This model exhibits distinct behavior patterns under extremely low and high levels of mechanical force. Under low force conditions, the substance exhibits solid-like characteristics and necessitates a specific degree of force to initiate flow. Conversely, under high force conditions, the substance behaves akin to a conventionally viscous fluid, known as Newtonian fluid, with a consistent viscosity [25].

(12) $$\tau ={\left(\sqrt{\tau_{\mathrm{yield},2}}+\sqrt{\eta \dot{\gamma }}\right)}^{2}$$

The variables $\tau_{\mathrm{yield},2}$ and η represent the initial shear stress and Newtonian viscosity, respectively.

#### 2.6.3 Power law model

The power law model is highly regarded for its simplicity and impressive accuracy within certain shear rate ranges. It is applicable to a wide range of fluids, both Newtonian and non-Newtonian, including those with shear thinning and shear thickening behaviors. As a result, researchers from various fields commonly use this model [6].

(13) $$\tau =m{\dot{\gamma }}^{n}$$

The flow index, denoted by the variable n, is a key factor in this model. In this study, we utilized the software Origin Pro to analyze viscosity data. Our focus was on achieving precise and reliable fitting of the experimental data.

### 2.7 Gelatinization kinetics

The process of gelatinization of starch is a complicated one, as it involves the disruption of hydrogen bonds between starch molecules and the uptake of water. The rate at which this gelatinization reaction occurs is influenced by the reaction kinetics. In particular, the reaction kinetics of gelatinization can be described using nth order reaction kinetics, where the rate of the reaction is directly proportional to the concentration of reactants raised to the power of n. It is assumed that this assumption holds true for modeling the reaction rate of gelatinization kinetics [26].

(14) $$\dot{\alpha }=A{\left(1-\alpha \right)}^{n}$$

$\dot{\alpha }$, is the rate of gelatinization determined by reaction rate (*A*), and *n* represents the order of kinetics.

Gelatinization in starch involves a change in enthalpy, which measures the total heat content of a system. As starch granules absorb heat energy, enthalpy increases. Analyzing changes in enthalpy during gelatinization provides valuable information about the energy associated with the process and starch sample characteristics.

The *α* parameter is significant in chemistry and materials science, helping to understand how heat energy is distributed and absorbed by starch molecules during gelatinization. Δ*H_P_* represents the specific energy required for modifying the starch structure, such as breaking hydrogen bonds and releasing water molecules. Conversely, Δ*H_T_* encompasses the overall energy exchange, including heat absorbed to raise the system's temperature [27]. The progression of gelatinization is represented by α, the ratio of Δ*H_p_* to Δ*H_T_*, according to the equation *α =* Δ*H_p_ /* Δ*H_T_*.

The speed at which a chemical reaction occurs is measured by the reaction rate constant, denoted as *A*, in chemical kinetics. In the Arrhenius model, *A* is influenced by the activation energy barrier associated with the reaction.

According to the Arrhenius model, for a reaction to occur, the molecules of the initial substances must overcome a specific energy barrier known as the activation energy. This barrier represents the minimum amount of energy required for the molecules to undergo the necessary changes and produce the desired end products.

In the case of starch gelatinization, the activation energy represents the energy needed to break the hydrogen bonds between starch molecules and allow water to enter the granule structure. By determining the activation energy of the gelatinization reaction, valuable information about the ease and speed at which starch can undergo gelatinization can be obtained. Higher activation energies indicate a more challenging and slower gelatinization process.

The reaction rate constant, A, is defined as the constant that relates the rate of the reaction to the concentration of the initial substances.

(15) $$A=A_{0}e^{-E_{a}/RT}$$

The regression method is employed to ascertain the reaction parameters, specifically $A_{0}$, $E_{a}$, and *n*.

### 2.8 Numerical considerations

The COMSOL Multiphysics® 6.1 software is utilized to conduct numerical simulations of the acoustic and velocity fields. Initially, the acoustic pressure field is determined, and the resulting data is employed to calculate the streaming force required for the determination of the velocity field. Additionally, the influence of gravitational acceleration is considered to account for the hydrostatic pressure distribution within the fluid container. Due to the very low magnitude of the velocities involved, the assumption of laminar flow is incorporated. The acoustic pressure field is obtained in the frequency domain, while the computational fluid dynamics simulation is carried out in a steady state.

The solution to the Keller-Miksis equation for modeling bubble dynamics is obtained using the ODE15s function of MATLAB (R2020b). This specific function is designed to solve stiff differential equations with a high degree of accuracy.

Operational Conditions:

### 2.9 Computational grids

**Fig. 2:**
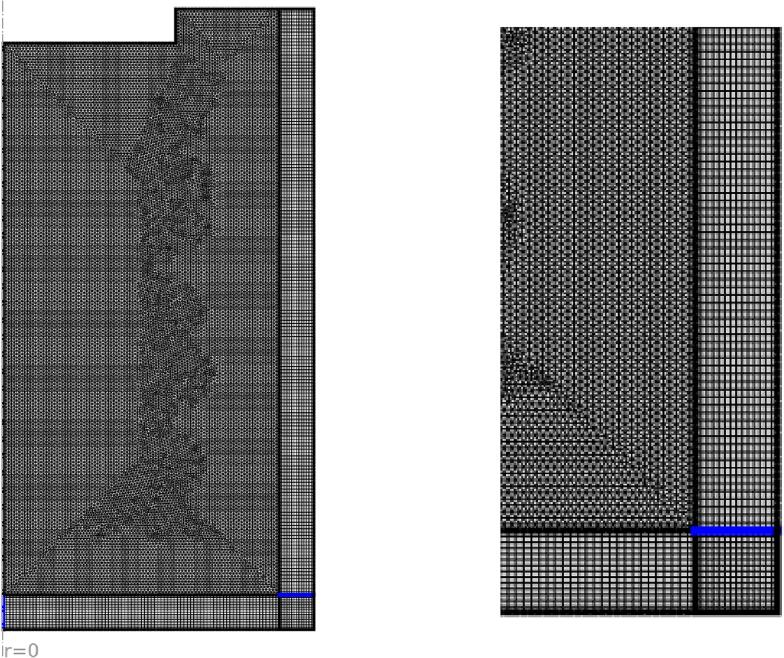
Schematic of the computational domain: a) entire domain, b) zoomed view.

A uniform triangular grid is utilized for the entire computational domain, except for the PML (perfectly matched layer) region where mapped rectangular cells are employed. Through a comprehensive grid study, it has been determined that the optimal number of grid cells is 101,483. The schematic diagram of the implemented grid is shown in Fig. 2.

## 3 Results and discussions

### 3.1 Numerical simulation data

**Fig. 3:**
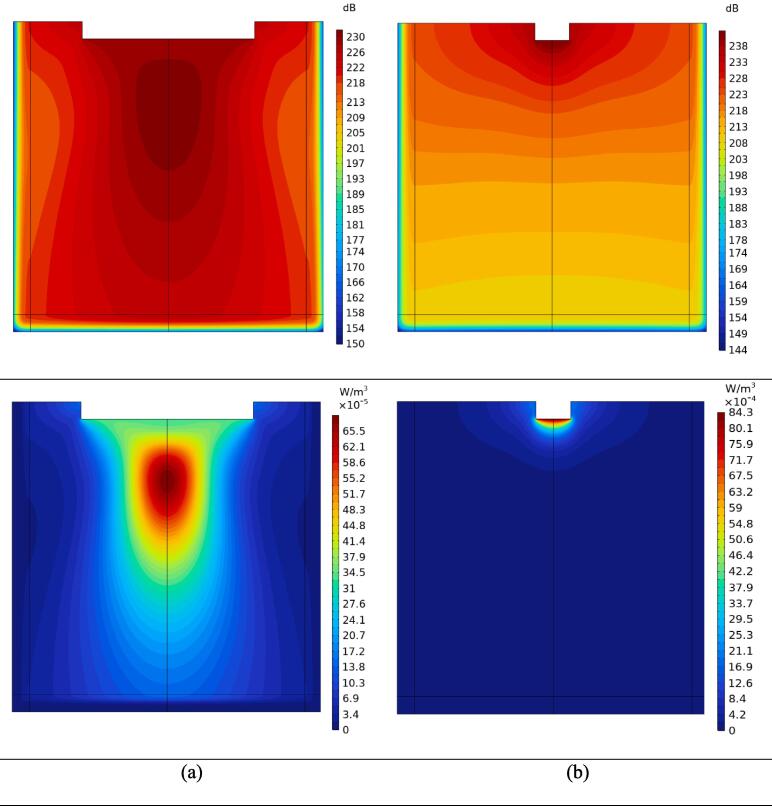
Total ultrasonic pressure (A), total sound pressure level (B), and contours of thermal power dissipation density (C) for probe tip diameter of a) 100 mm and b) 20 mm.

Fig. 3-A illustrates the total ultrasonic pressure for both small and large probes operating at a frequency of 24 kHz and power of 400 W. Increasing the probe diameter results in a reduction of ultrasonic intensity and consequently, a decrease in the applied acoustic pressure amplitude. Specifically, when the probe diameter is increased from 20 to 100 mm, the pressure amplitude is reduced by 73.2%. However, the larger probe area allows for a larger portion of the top surface to be exposed to oscillating pressure waves, creating a more uniform pressure pattern within the domain. In contrast, the smaller 20 mm probe releases more focused ultrasonic waves in a smaller area, leading to more rapid attenuation as the waves pass through the medium. This can be observed in the 89.5% reduction in pressure amplitude between the compression waves in the top and bottom regions for the smaller probe, compared to the 24% reduction for the larger probe. As expected, the pressure amplitude in the perfectly matched layer (PML) region decreases rapidly.

In Fig. 3-B, the total sound pressure level is shown for both examined probes. With a 25-fold increase in probe area, the pressure level is reduced from 241.7 to 231.2 dB. Additionally, at each height level, the maximum pressure level occurs at the center line of the container. For the 20 mm probe diameter, the highest-pressure level is found in the vicinity of the horn tip and gradually decreases with depth. Conversely, for the 100 mm diameter probe, the maximum pressure level is located approximately 4 cm below the horn tip surface.

Fig. 3-C displays the contours of thermal power dissipation density. This dissipated power, calculated per unit volume, significantly affects the determination of thermal energy delivered to the fluid. Variation in probe size leads to significant changes in the magnitude and pattern of dissipated power. The maximum dissipated power decreases from 8.43 to 0.655 mW/m^3^ with an increase in probe diameter from 20 to 100 mm. Furthermore, for the smaller transducer, the dissipated power is highly concentrated near the horn tip, while a more uniform and extended heated area is observed for the larger probe.

**Fig. 4:**
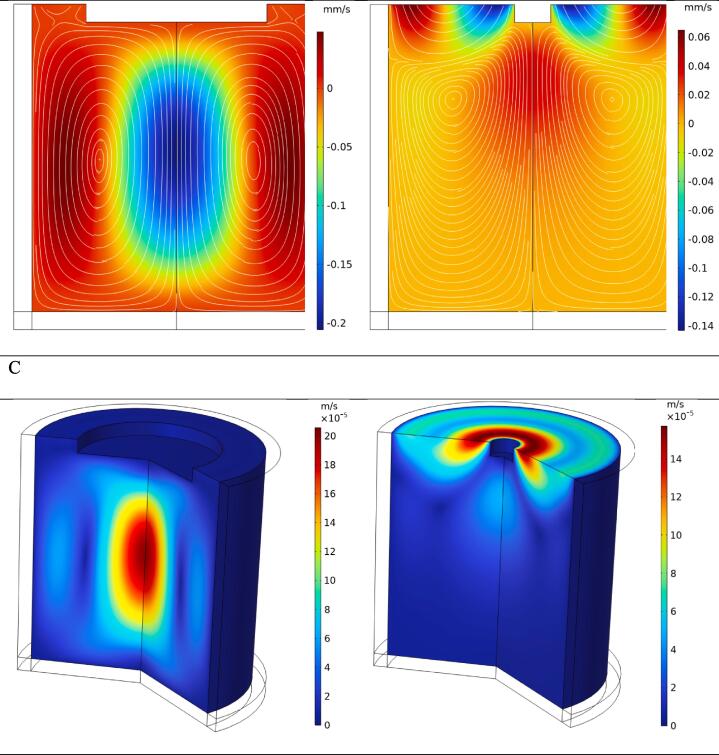

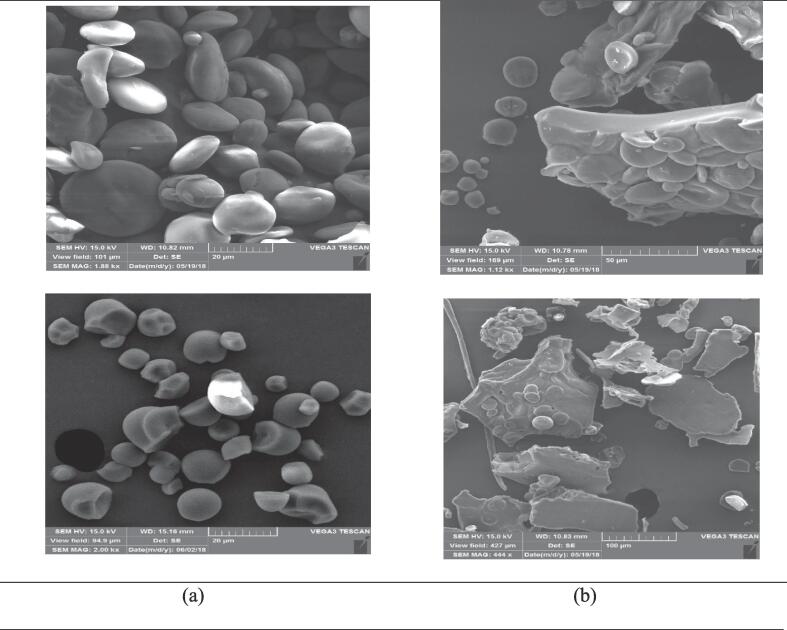
Contours of streaming force applied to the fluid (A), the vertical component of the velocity field (B), the velocity magnitude (C) and D) micrographs for probe tip diameter of a) 100 mm and b) 20 mm.

The streaming force applied to the fluid, resulting from fluctuating acoustic pressure, greatly influences flow streaming. The contours of the streaming force in Fig. 4-A indicate that increasing the probe diameter from 20 to 100 mm reduces the maximum magnitude of the streaming force by over 10 times (from 29.2 to 2.51 mN/m^3^). Furthermore, the domain affected by this streaming force is more restricted for the 20 mm diameter probe compared to the 100 mm probe. Consequently, the fluid experiences a highly concentrated volumetric force in a small volume with rapid reduction in farther regions for the smaller probe. The impact of this force can be better visualized through examination of the velocity contours.

Fig. 4-B and C demonstrate the motion of the fluid and the velocity field components. Fig. 4-B displays the vertical component of the velocity field and the associated streamlines for both probe configurations. With the larger probe (100 mm diameter), a downward flow is created due to the application of a streaming force over a larger area. The maximum downward velocity occurs at the middle depth of the fluid container, measuring approximately 0.2 mm/s. In contrast, the smaller probe results in a more intricate flow pattern, with the highest downward velocity around 0.14 mm/s observed along the peripheral walls of the transducer. Additionally, the larger probe configuration generates dominant flow circulations, while the smaller probe configuration divides the fluid domain into small and large circulations near the top and bottom surfaces, respectively.

Fig. 4-C also depicts the contours of the velocity magnitude at a sectioned view of the fluid container. When using a 100 mm diameter tip, the flow is confined below the horn tip due to the large tip area, resulting in a bulk downward flow followed by upward circulation along the peripheral container’s walls. Conversely, with a 20 mm diameter, two factors contribute to a more complex flow pattern: the presence of very intense streaming force near the horn tip and increased space for the fluid to occupy. The highest velocity magnitude occurs at the flow surface around the probe.

To determine the optimal probe size for ultrasound treatment in food processing, one must consider the specific effects of probe size on thermal power dissipation and fluid dynamics. The study’s data clearly indicate that a smaller probe diameter, such as 20 mm, results in higher ultrasonic intensity and pressure amplitude, which are conducive to more efficient gelatinization, as evidenced by the significant reduction in reaction enthalpy and Gibbs free energy for both wheat and tapioca starches.

Conversely, a larger probe diameter, such as 100 mm, leads to a decrease in ultrasonic intensity and pressure amplitude, which results in a more uniform pressure distribution within the fluid. This is advantageous for processes that require consistent treatment across a larger surface area. The reduced streaming force with larger probes also suggests that they may be preferable for the treatment of delicate materials where minimal mechanical disruption is desired.

In summary, the selection of an appropriate probe size is guided by the desired balance between gelatinization efficiency and the uniformity of treatment. Smaller probes are more effective for rapid and intense gelatinization, while larger probes are better suited for uniform treatment over larger areas with less mechanical impact on the material.

### 3.2 Microstructure

Fig. 4-D displays micrographs of NWS, NTS, UPWS, and UPTS. Consistent with previous literature findings [28], NWS exhibited characteristic A-type starch granules with a lenticular shape and larger size ranging from 20-50 μm. Additionally, NWS contained B-type starch granules with a spherical shape and smaller size ranging from 2-10 μm. On the other hand, NTS displayed larger flake- and irregularly shaped granules ranging from 3- 32 μm [16], [29]. Observations on the pre-gelatinized starch treated with the small probe (20 mm diameter) revealed several structural changes. Firstly, there was a significant reduction in granule size, accompanied by the presence of depressions, which were more pronounced in wheat starch compared to tapioca starch. Secondly, indentations were observed on the surface of the tapioca starch granules. Thirdly, there was the separation of granular masses from each other, resulting from the breakdown of inter- and intra-granule forces. Lastly, the formation of granular fractures and cracks was observed in both wheat and tapioca starch samples. The susceptibility of starch to ultrasonication has been associated with various factors, including starch type, composition, and concentration in the slurry, as well as the frequency, energy input, temperature, and treatment time of the system [30]. When subjected to ultrasound treatment, starch experiences fractures and mechanical damage due to the collapse of cavitation bubbles, micro-jetting, and the presence of high local velocities of liquid layers near the starch granules. These phenomena generate local shear forces that lead to the breakage of polymer chains and damage to the granules. Additionally, during the collapse of cavitation bubbles, water molecules undergo partial degradation, resulting in the formation of OH and H radicals. These radicals possess high reactivity and can influence the chemical and physical properties of the starch [1].

The micrographs obtained for ultrasound-assisted pre-gelatinized starch (using a probe diameter of 100 mm) in Fig. 4-D showed that the treatment resulted in the destruction of the granular structure of native starch. Instead, the starch transformed into a sheet-like structure with air bubbles dispersed within the continuous solid phase. This observation suggests that the ultrasound treatment led to the gelatinization of all starch granules, regardless of the plant species they originated from. Gelatinization refers to the process in which starch granules lose their crystalline structure and absorb water, resulting in the formation of a viscous gel-like substance. The destruction of the granular structure and the formation of a sheet-like structure with air bubbles indicate that the starch underwent gelatinization during the ultrasound treatment.

### 3.3 Effect of probe size geometry on the reaction kinetics and thermodynamic data analysis

**Fig. 5:**
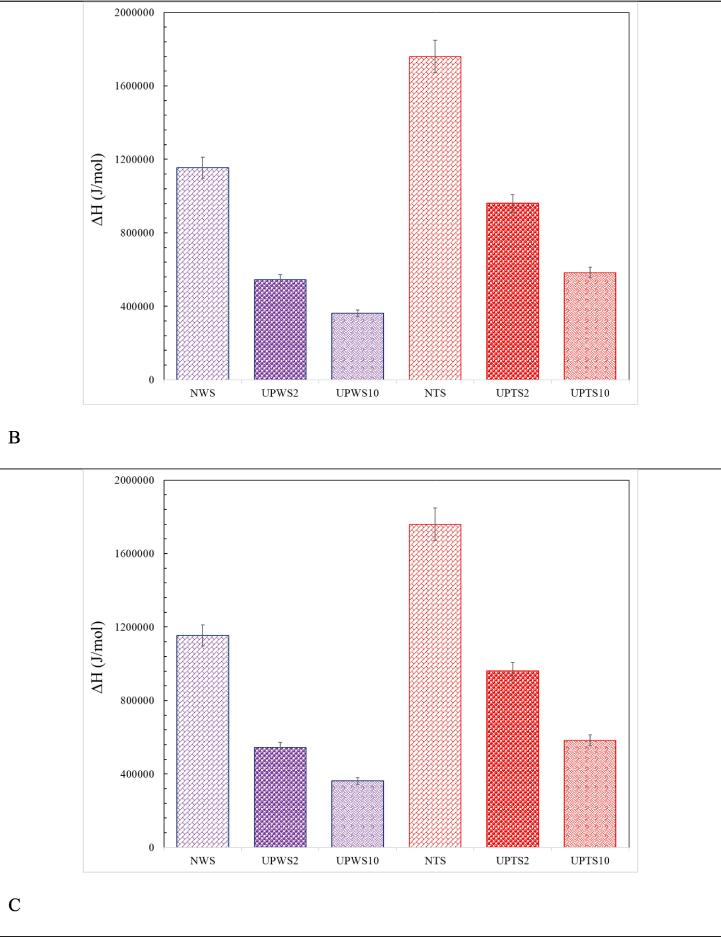

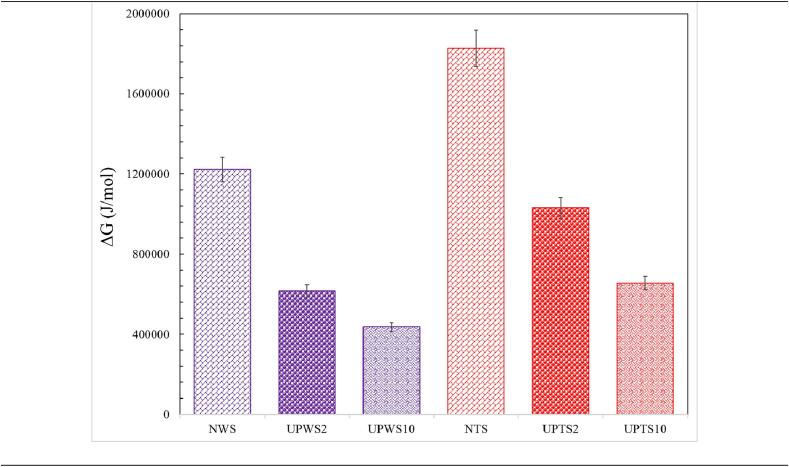
The magnitude of enthalpy changes (A), entropy changes (B) and Gibbs energy (C) during gelatinization for various treatments.

Fig. 5 depict the changes in various thermodynamic properties, such as enthalpy, entropy, and Gibbs energy, for both types of starch and different treatments. These properties provide valuable information about the nature of the process.

The gelatinization process, shown in Fig. 5-A, is affected by the energy required for the reaction. A positive ΔH value indicates an endothermic reaction, meaning energy is needed for the reaction to progress. The results show that sonication treatment significantly reduces the amount of energy required for both types of starch. Understanding the scientific justifications behind these outcomes is crucial. Sonication treatment employs ultrasonic waves for the purpose of particle breakdown and homogenization enhancement [31]. The ΔH values primarily indicate the loss of double-helical structure rather than the loss of crystalline order during gelatinization. The decrease in ΔH values of ultrasound-assisted pre-gelatinized starch can be attributed to the disruption of double helices present in the non-crystalline and crystalline regions of the granules, leading to a reduction in energy consumption and the enhanced efficiency of the gelatinization process. These thermal results are consistent with previous studies on ultrasound-assisted gelatinization of starch by [32] and [2].

Additionally, as the probe diameter increases, the required thermal energy decreases even further. For example, using a 20 mm and 100 mm probe leads to a reduction of 52.7 % and 68.6 % in reaction enthalpy for wheat starch compared to native starch, respectively. Similar reductions of 45.4 % and 66.8 % are observed for tapioca starch. The larger probe is more effective in delivering energy to the starch mixture, resulting in a greater reduction in enthalpy change during the gelatinization process.

Entropy refers to the extent of disorder or randomness within a system. In the context of the gelatinization process, starch undergoes water absorption and expansion, resulting in the breakdown of its granules and the disturbance of their organized arrangement. As a consequence, the starch molecules experience an elevated level of disorder, which consequently alters the entropy. Sonication treatment also affects the entropy variations, as shown by the ΔS parameter in Fig. 5-B. The implementation of sonication increases the entropy for both wheat and tapioca starches, and this increase is directly related to the size of the probe used. Specifically, using a 20 mm probe and a 100 mm probe leads to entropy increases of 2.56 % and 4.72 % for tapioca starch, and 3.11 % and 4.97 % for wheat starch, respectively.

The Gibbs energy is an inherent characteristic of a system in thermodynamics, quantifying the energy accessible within the system for performing work under constant temperature and pressure conditions. As a derived quantity, it is determined by taking the difference between the enthalpy change of a reaction and the product of the temperature and the entropy change associated with that reaction. A negative value of Gibbs energy signifies the spontaneous nature of the reaction, enabling it to proceed in the forward direction. Conversely, a positive value denotes a non-spontaneous reaction, implying that it will not occur spontaneously unless energy is supplied to the system. The results indicate that sonication treatment has a favorable effect on reducing the positive ΔG value [33]. This means the gelatinization reaction becomes less non-spontaneous and requires less energy to progress. However, the magnitude of this effect depends on the size of the probe used. By incorporating a 20 mm probe and a 100 mm probe, the ΔG value is lowered by 45.9 % and 64.3 % for wheat starch, and 43.6 % and 64.1 % for tapioca starch, respectively (Fig. 5-C).

Overall, the reduction in Gibbs energy through sonication treatment can be interpreted as follows: firstly, it increases temperature, weakening intermolecular forces and reducing the energy needed for gelatinization. Secondly, it enhances mass transfer by creating microstreaming and turbulence, facilitating faster water penetration into the starch granules and further reducing the energy required for gelatinization. Finally, sonication disrupts the molecular structure of the starch, leading to swelling and gelatinization, which again reduces the energy needed for gelatinization and decreases the variability of Gibbs energy.

### 3.4 Effect of probe size geometry on ultrasonicated starches’ rheology modeling

For both wheat and tapioca starch, the pre-gelatinization process resulted in a significant increase in peak viscosity and breakdown viscosity values. Meanwhile, the pre-gelatinized samples obtained through ultrasonication with the larger probe (100 mm) exhibited enhanced viscosity at cold temperatures (<50 °C) compared to those generated using the smaller probe (20 mm). This indicates that the starch underwent a higher degree of swelling and gel formation during the pre-gelatinization process. Additionally, the pasting temperature, as well as the final viscosity values, were significantly decreased after pre-gelatinization. This suggests that the starch underwent a more rapid gelatinization and had a reduced ability to retrograde or form a stable gel after cooling.

The order of viscosity superiority was observed as UPWS10 > UPTS10 > UPWS2 > UPTS2. A significantly higher peak viscosity (p < 0.05) was observed for UPWS and UPTS treated with the larger probe (100 mm diameter) compared to the smaller probe (20 mm diameter), with UPWS showing significantly higher values than UPTS. Ultrasonication employing the larger probe (100 mm diameter) readily disrupted the molecular structure of starch, including intra- and intermolecular hydrogen bonds within the starch chains. The mobility of starch chains becomes more pronounced as the temperature rises, facilitating the infiltration of water molecules into pre-gelatinized granules and resulting in the release of some amylose molecules from within the starch granules and gelation process of leached amylose on the surface of the granules [34], [35]. This finding is consistent with previous research [36] that ultrasound has a more pronounced effect on the pasting behavior of high-amylose starch (wheat) compared to low-amylose starch (tapioca).

**Fig. 6:**
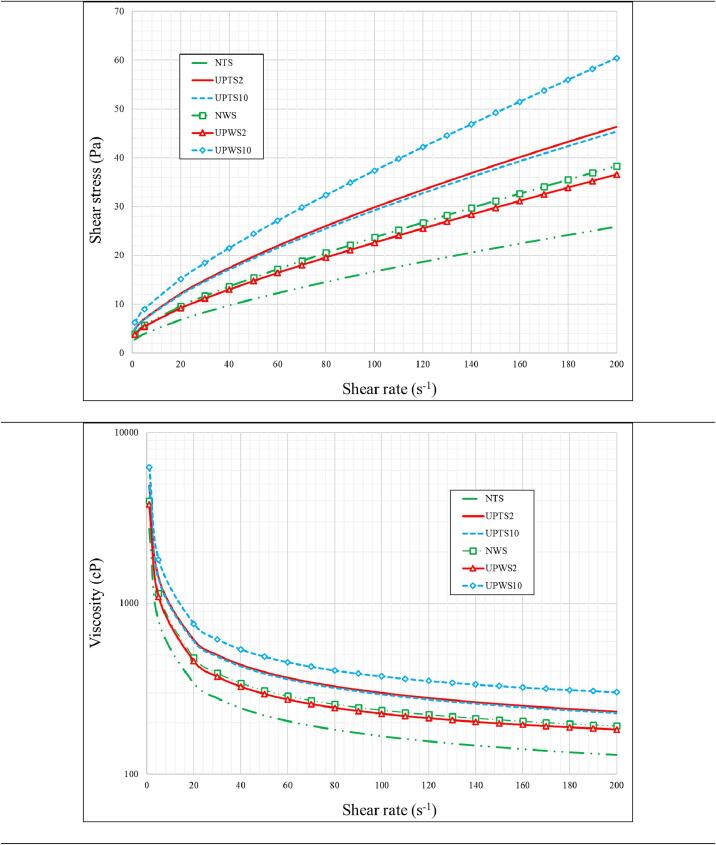
The relationship between shear stress and shear rate (A) and viscosity and shear rate (B) under different operational conditions.

Fig. 6 illustrates the relationship between shear stress and shear rate under different operational conditions. At very low shear rates, a residual shear stress exists, indicating the presence of the yield shear stress that must be considered. The yield shear stress is the stress required to initiate the fluid motion. Furthermore, the relationship between shear stress and shear rate is non-linear, indicating non-Newtonian flow behavior.

When considering the effect of starch type and probe diameter, several observations are made. For tapioca starch, sonication increases the required shear stress compared to native starch. The effect of probe size is negligible, with a difference of less than 1.6 % between shear stresses for 20 mm and 100 mm diameter probes [37]. However, for wheat starch, using a 100 mm diameter probe significantly increases the required shear stress, while there is no significant difference between native starch and sonication with a 20 mm diameter probe (Fig. 6-A).

A similar discussion can be done for the variation of viscosity with shear rate. Fig. 6-B shows how viscosity in *cP* varies with shear rate for all cases. Generally, viscosity decreases with shear rate, indicating shear thinning behavior of the fluid flow.

For tapioca starch, sonication treatment can increase viscosity by up to 75.5 % compared to native starch, regardless of probe diameter. However, for wheat starch, only using a 100 mm diameter probe has a significant impact on viscosity variations compared to the native starch case, with an increase of up to 57.7 %.

**Fig. 7:**
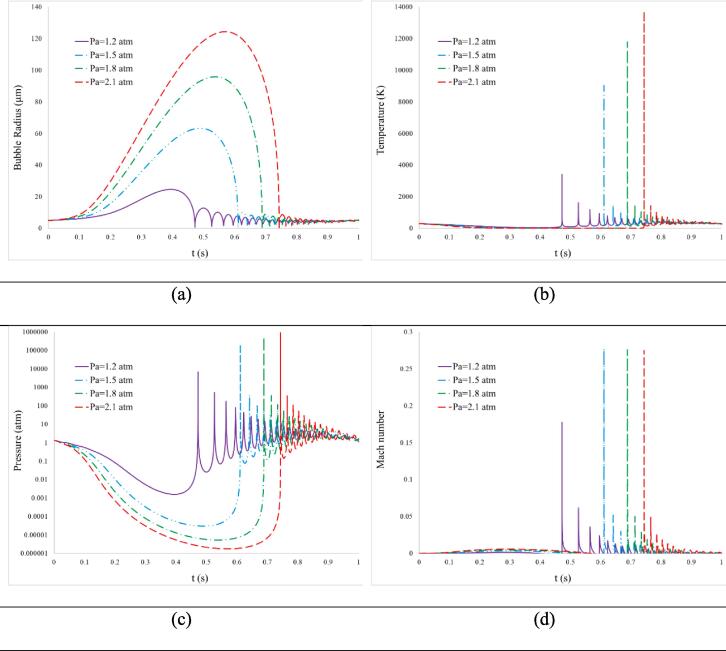
The time history of parameters variation during bubble dynamics: a) Radius, b) Temperature, c) Pressure and d) Mach number.

The impact of liquid pressure amplitude on the dynamics of bubbles is shown in Fig. 7 over time. The data extracted from the Keller-Miksis equation and thermodynamic modeling includes the radius, temperature, pressure, and Mach number of the bubbles.

The results indicate that as the pressure amplitude increases, the initial burst of the bubbles occurs earlier and the radius at which the explosion happens also increases. Specifically, increasing the pressure amplitude from 1.2 to 2.1 atm results in the burst bubble radius increasing from 24.65 to 124.23 μm, and the explosion occurring later, from 0.394 to 0.562 s (Fig. 7-a).

When the amplitude of fluid pressure increases, it creates higher pressure gradients within the fluid. These increased gradients cause microbubbles to undergo more extensive expansion and contraction cycles with each acoustic wave. This phenomenon, known as acoustic cavitation, refers to the response of bubbles to pressure changes [38]. During expansion, the bubble size increases, while during compression, it decreases. As pressure amplitude rises, the bubbles experience more pronounced expansion and contraction, leading to larger variations in their sizes.

At certain pressure amplitudes, the bubbles reach a critical size during expansion where the surrounding fluid can no longer support the increased surface area. As a result, the microbubbles are unable to withstand the pressure difference and rupture or burst. This process is called inertial cavitation. The increase in bubble radius at the explosion instance significantly affects the pressure and temperature release during the burst, which will be discussed further.

The pressure and temperature of the bubbles during subsequent expansion and compression cycles are illustrated in Fig. 7-b and c. Increasing the pressure amplitude from 1.2 to 2.1 atm leads to an increase in pressure and temperature at the most contracted state, from 3408.8 K and 6823.7 atm to 13715.1 K and 916139.6 atm, respectively.

With higher pressure amplitudes, the bubble undergoes more intense compression, resulting in the storage of a greater amount of potential energy. When the bubble reaches maximum compression and begins to expand again during the rarefaction phase of the sound wave, this stored potential energy is rapidly converted into kinetic energy.

The rapid expansion of the bubble during the rarefaction phase causes a forceful collapse, known as cavitation. This collapse generates extremely high temperatures and pressures within a small area of the bubble. The temperature increase is a consequence of adiabatic compression, where the sudden reduction in volume during collapse leads to a temperature rise.

Therefore, higher pressure amplitudes result in an increased input of energy into the bubble during compression, leading to more forceful collapses and higher temperatures and pressures at the point of microbubble explosion. Additionally, the Mach number of the bubble's deformation cycles, as shown in Fig. 7-d, increases with higher pressure amplitudes.

The gelatinization process can be considered as a reaction that proceeds with a finite reaction rate. This rate is influenced by the activation energy (E_a_) and temperature. Activation energy can be understood as an energy barrier that must be overcome for the reaction to progress. Higher activation energy values indicate that a higher temperature is required for the reaction to reach a specific level of progress (Table 1).

**Table 1:** The kinetic data of the gelatinization process for all tested cases.

	***E_a_***	***A_0_***	***n***	***R^2^***	***Adj-R^2^***	***RMSE***
**NWS**	1.16 × 10^6^	421.5	0.8706	0.8741	0.8644	0.3909
**UPWS2**	5.49 × 10^5^	199.1	0.8556	0.8538	0.8425	0.4212
**UPWS10**	3.65 × 10^5^	129.6	0.8951	0.9177	0.9114	0.3026
**NTS**	1.76 × 10^5^	635.1	0.8632	0.8669	0.8567	0.4018
**UPTS2**	9.63 × 10^5^	345.6	0.9313	0.9137	0.9071	0.3235
**UPTS10**	5.87 × 10^5^	208.4	0.9452	0.9298	0.9244	0.2919

The kinetic data of the gelatinization process for all tested cases are presented in Table 1. The results suggest that the effects of sonication and probe size differ for wheat and tapioca starches. Sonication decreases the activation energy for wheat starch, and the use of a larger probe further reduces this energy barrier. In contrast, ultrasonic transducer application increases the activation energy for tapioca starch. However, similar to wheat starch, increasing the probe size from 20 to 100 mm reduces the activation energy for tapioca starch. Therefore, the utilization of a larger probe decreases the temperature at which gelatinization begins [39].

The reaction rate falls within the range of 0.8556 to 0.9452, indicating an acceptable precision for a first-order reaction rate. A first-order reaction rate implies a stronger dependence on the reactant concentration compared to other factors, such as temperature. Additionally, the statistical assessment parameters suggest that the model accurately predicts the progress of the reaction.

The rheology modeling is conducted using three popular models: the power law, Casson, and Herschel-Bulkley models. These models are chosen to encompass different aspects of flow behavior, as each model considers specific rheological parameters.

The data extracted for the Herschel-Bulkley model is presented in Table 2. The results indicate that a finite shear yield stress ($\tau_{\mathrm{yield},1}$) is obtained for all tested cases, which aligns with the findings in Fig. 6. The yield shear stress increases with sonication for both types of starch and probe sizes. The flow index parameter (*n*) ranges from 0.6746 to 0.7372 (with an average of 0.704) for wheat starch, and from 0.7605 to 0.7862 (with an average of 0.774) for tapioca starch. Since the flow index is below unity, the shear thinning behavior is justified. However, it is observed that native and sonicated wheat starch exhibit a higher degree of shear thinning behavior. Additionally, the Herschel-Bulkley model demonstrates high accuracy based on the statistical assessment data.

**Table 2:** The Herschel-Bulkley, power-law, and Casson model parameters.

Herschel-Bulkley model						
	***α***	***β***	$\tau_{yield,1}$	***R^2^***	***Adj-R^2^***	***RMSE***
**NWS**	0.4796	0.7372	2.395	0.9947	0.9941	0.5424
**UPWS2**	1.035	0.6992	4.01	0.9947	0.9941	0.9438
**UPWS10**	1.166	0.6746	3.049	0.9945	0.9938	0.9482
**NTS**	0.5688	0.7753	3.699	0.9976	0.9973	0.5381
**UPTS2**	0.5065	0.7862	3.699	0.9965	0.9961	0.6115
**UPTS10**	0.9629	0.7605	5.557	0.995	0.9945	1.202

Power-law model						
	***m***	***n***	***R^2^***	***Adj-R^2^***	***RMSE***	
**NWS**	1.054	0.6036	0.989	0.9884	0.7616	
**UPWS2**	2.102	0.5799	0.9899	0.9893	1.274	
**UPWS10**	1.988	0.5852	0.9915	0.9911	1.143	
**NTS**	1.367	0.6255	0.9906	0.9901	1.028	
**UPTS2**	1.293	0.6295	0.9887	0.9881	1.069	
**UPTS10**	2.199	0.6199	0.9887	0.9881	1.764	

Casson model						
	**η**	$\tau_{yield,1}$	***R^2^***	***Adj-R^2^***	***RMSE***	
**NWS**	0.06311	2.347	0.9934	0.993	0.5716	
**UPWS2**	0.1239	3.735	0.9963	0.9961	0.8028	
**UPWS10**	0.1139	3.994	0.9953	0.9951	0.8538	
**NTS**	0.1086	2.532	0.9963	0.9961	0.668	
**UPTS2**	0.09731	2.639	0.9947	0.9945	0.7316	
**UPTS10**	0.1634	4.251	0.9956	0.9953	1.117	

The parameters of the power law model, including the flow index and consistency parameters, are also listed in Table 2. As expected, the flow index is below one for all cases, with averages of 0.590 and 0.625 for wheat and tapioca starches, respectively. The obtained flow index values are lower than those for the Herschel-Bulkley model. This difference can be attributed to the exclusion of yield shear stress in the power law model. The effect of probe size on the power law model parameters is found to have a greater impact on the flow consistency parameter, while the flow index shows less variation.

Table 2 presents the parameters for the Casson model in all examined cases. The Casson model assumes the presence of a yield shear stress at very low shear rates, accompanied by nearly Newtonian behavior at higher shear rates. These assumptions align well with the observed data in Fig. 6, indicating that this model is suitable for rheology modeling in this study. The predicted yield shear stress increases with both sonication and probe size for both types of starch. The average yield shear stress values are calculated as 3.36 and 3.14 for wheat and tapioca starches, respectively. These values are lower than those predicted by the Herschel-Bulkley model, which can be attributed to the different mathematical formulations of these models.

To determine the best model for simulating the flow behavior, the average value of the R^2^ parameter is calculated. The Herschel-Bulkley, power law, and Casson models yield R^2^ values of 0.996, 0.990, and 0.995, respectively.

## 4 Conclusion

The study conclusively demonstrates that ultrasound probe size is a critical factor in starch gelatinization, influencing ultrasonic pressure, thermal power dissipation, and fluid dynamics. A smaller 20 mm probe significantly enhances the gelatinization efficiency for wheat starch, reducing reaction enthalpy by 52.7 % and Gibbs free energy (ΔG) by 45.9 %. For tapioca starch, the same probe achieves a 43.6 % reduction in ΔG. The larger 100 mm probe, while lowering ultrasonic intensity, ensures a more uniform pressure distribution, beneficial for consistent treatment across larger areas. This probe size also decreases the maximum dissipated power from 8.43 to 0.655 mW/m^3^ and reduces the pressure amplitude by 73.2 %.

Thermodynamically, the larger probe diameter leads to greater entropy increases, with tapioca starch showing a 4.72 % increase and wheat starch a 4.97 % increase, compared to 2.56 % and 3.11 %, respectively, with the smaller probe. The ΔG value further drops by 64.3 % for wheat starch and 64.1 % for tapioca starch with the 100 mm probe, underscoring the probe’s influence on the energy dynamics of the gelatinization reaction. Rheologically, both starches exhibit shear thinning behavior, fitting well with the Casson model, while the Power Law and Herschel-Bulkley models also accurately predict flow behavior, considering yield shear stress at low shear rates. The study also delves into bubble dynamics, noting that increasing the pressure amplitude from 1.2 to 2.1 atm causes the burst bubble radius to increase significantly, affecting the microstructure and efficiency of the starch gelatinization process.

In light of these findings, the appropriate probe size selection hinges on the specific requirements of the starch processing application. For high-efficiency gelatinization where rapid and significant structural disruption of starch granules is desired, the 20 mm probe is recommended. However, for processes that prioritize uniform treatment and are sensitive to mechanical impact, the 100 mm probe is the better choice due to its gentler and more evenly distributed effect. The study provides a clear directive for food processors: smaller probes are ideal for intense, efficient treatments, while larger probes should be used for uniform, large-surface applications. The decision ultimately rests on balancing the trade-offs between intensity and uniformity to achieve the desired outcome in starch processing.

## 5 Consent to Participate

The present paper has been approved by all named authors.

## 6 Consent for Publication

The present paper, which is original, has not been published before and is not currently being considered for publication elsewhere.

## Author contribution

Elahe Abedi and Reza Roohi wrote the manuscript, conceived and designed the research, and analyzed the data. Seyed Mohammad Bagher Hashemi and Masoud Akbari conceived and designed the research and analyzed the data. Elahe Abedi and Reza Roohi conducted the experiments. All the authors read and approved the manuscript.

## Funding statement

Not applicable.

## Ethical Approval

This article does not contain any studies with human participants or animals performed by any of the authors.

## CRediT authorship contribution statement

**Reza Roohi:** Writing – review & editing, Writing – original draft, Software, Formal analysis, Data curation. **Elahe Abedi:** Writing – review & editing, Writing – original draft, Supervision, Project administration, Methodology, Investigation. **Seyed Mohammad Bagher Hashemi:** Writing – original draft, Project administration, Methodology, Investigation. **Masoud Akbari:** Validation, Software, Data curation, Conceptualization.

## Declaration of competing interest

The authors declare that they have no known competing financial interests or personal relationships that could have appeared to influence the work reported in this paper.

## Data Availability

Research data are not shared.
